# Ink-Jet Printing: Detection of Liquid Distribution by 1D MRI

**DOI:** 10.3390/molecules31213817

**Published:** 2026-10-22

**Authors:** Eric Schmid, Rahel Lerner, Nele Weis, Mathias Stahlberger, Hermann Nirschl, Gisela Guthausen

**Affiliations:** 1 Institute of Mechanical Process Engineering and Mechanics, Karlsruhe Institute of Technology, 76131 Karlsruhe, Germany; 2 Chair of Water Chemistry and Water Technology, Engler-Bunte-Institut, Karlsruhe Institute of Technology, 76131 Karlsruhe, Germany

**Keywords:** NMR, MRI, ink-jet printing, pore size distribution of paper

## Abstract

Ink-jet printing applying water-based inks currently attracts more attention also due to ecologic reasons. Problems occur during storage and transport of printed paper due to the liquid distribution within the paper or carton board. NMR spectroscopy and µCT were adapted and applied to investigate inks, papers and carton boards. The components already show a large variability in their properties which becomes even more important when investigating them together, in combination, in a pseudo-printing process, for example, by MRI. The process of liquid dispersion in the paper substrate was investigated, time-resolved with one-dimensional spatial resolution along the printing direction, which allowed us to study penetration and spread of the fluid in the substrate. The focus of this paper is on the methodical basis of this process. The experiments reveal technically relevant parameters like chemical composition of inks, pore size distribution of the paper, and kinetics of ink penetration and distribution in combination of both, but also show the multitude of influencing parameters in printing processes.

## 1. Introduction

In ink-jet printing of aqueous inks, open questions concern wettability of substrates and separation of pigments and liquids as well as printing quality. The spread of liquids is highly dynamic and broad after dropping very small droplets onto the substrate with high-speed print headers; subsequent drying determines printing quality and stability during storage and transport, where temperature variations may lead to agglutination. Monitoring of liquid distribution in paper or carton boards, referred to in the following as substrate, is therefore highly desired. Monitoring should be fast, accurate, and non-destructive, such that longitudinal studies are feasible; the focus of this paper is therefore on this aspect of research. Established methods often characterize the printing result at the substrate’s surface, while non-destructive characterization within the substrate itself is often not achievable. The approach in this paper is to use high-field MRI in its one-dimensional form of spatial resolution (1D profiling) along the axis of dropping onto and penetration into the paper. This approach has already been realized at low magnetic fields using NMR based on permanent magnets [1,2,3,4], which is a technologically different approach: NMR hardware, data acquisition, and processing differ significantly. The investigated materials are also different. Ultrafast imaging using an OW4 sequence [5] was applied to study liquid penetration into nylon and polyvinylidene fluoride sheets by a single-sided NMR device, with a temporal resolution of 10 ms and a 1D spatial resolution of 14.5–18 μm. Penetration of liquids, which often contained paramagnetic substances, and subsequent drying were studied via characterization of the liquids’ front and signal amplitudes.

Apart from the printing process and the printing result, the single components involved in printing, i.e., substrates and inks, should be well-known [6]. ^1^H NMR spectroscopy was applied to reveal the main chemical composition of model inks with recipes close to commercial inks in inkjet printing. The characterization of the fibrous structure of market and experimental substrates was made by micro-computer tomography (µCT) with a sufficiently good spatial resolution to map the fibers and their networks [7]. This approach allows for the calculation of pore size distributions which are represented by the equivalent diameter *d*_eq_. This is due to the open pore structure of these fiber networks.

Starting with the characterization of the single-component paper (µCT) and ink (NMR spectroscopy), experiments will follow to resolve penetration and spreading of ink in the substrates which perspectively allows insights into interactions between ink and substrate [8] with a focus on extracting characterizing measures usable in paper and ink development in the future.

## 2. Results

### 2.1. Paper Structure: µCT

The structure of paper substrates is determined by fibers which form a network with the well-known macroscopic stability. Apart from surface properties which are modified by dedicated coating, pore sizes and their distribution co-determine the printing properties including the ink’s spreading and drying apart from mechanical stability. These parameters are therefore of interest for paper characterization within the context of ink penetration.

**Figure 1:**
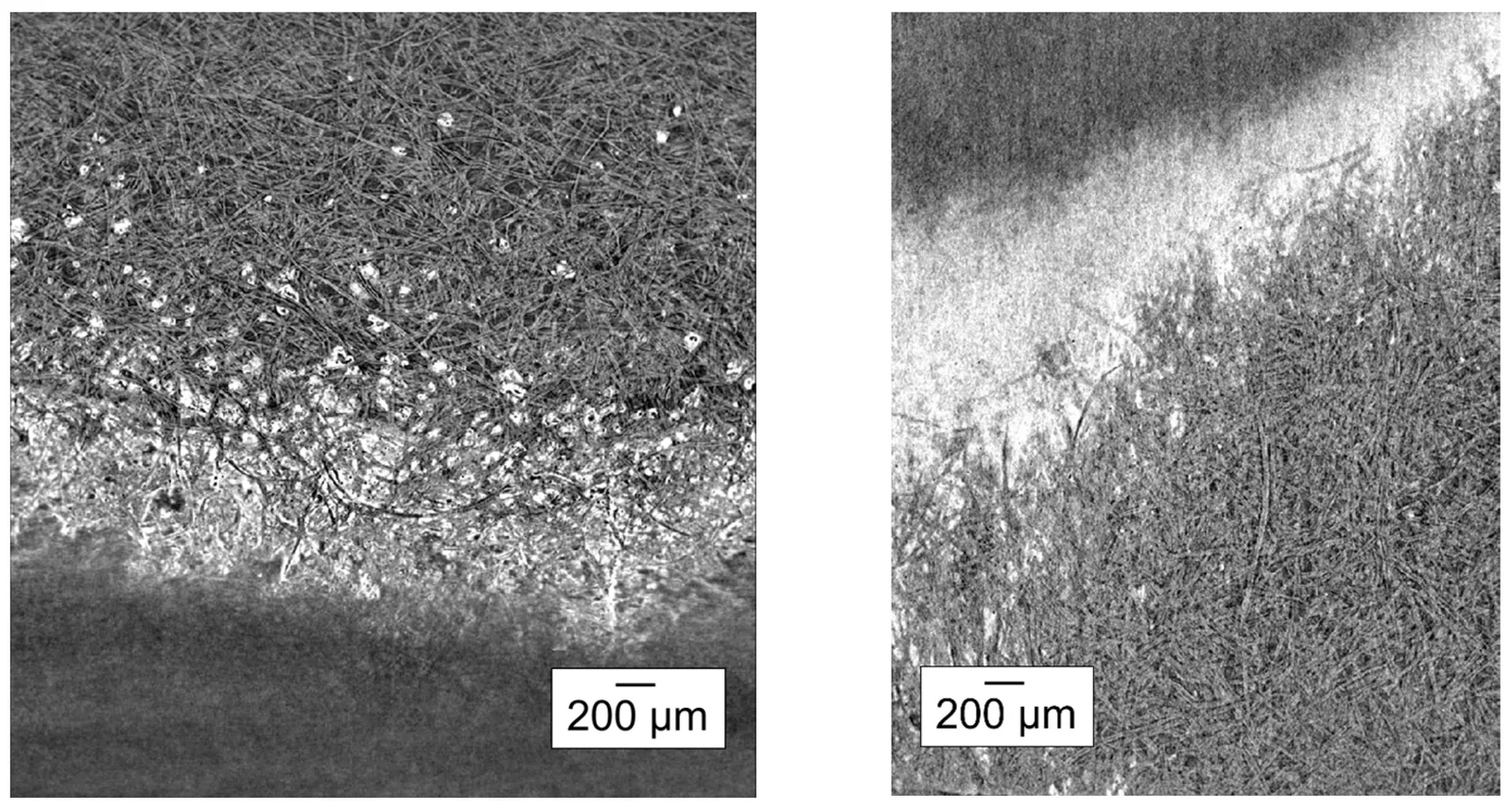
µCT images on two paper samples. Fiber structures, surface coatings (white areas), and particulate components (white spots in the inner of the paper) differ significantly, thereby establishing the metrological basis for quantitative image analysis.

3D µCT images of commercially available papers and carton boards were measured and analyzed following the procedure described in Section 4, Materials and Methods. The papers showed a divergent microscopic fine structure which can be attributed to the fibers themselves as well as to paper processing. The surface coating of the papers distinctly appears in the µCT images (Figure 1).

Numerical image analysis led to the pore size distributions which characterize, first of all, the measured, actual section of the papers, but are representative for the type of paper as it is rather uniform in the lateral dimensions. The mask’s volume was placed in the middle of the paper section to minimize the influence of layered structures or surface coatings. The classification scheme of the histogram was chosen such that the smallest bin was around 5 µm, taking into account the chosen spatial resolution of the µCT measurements. The histograms were finally modelled by log-norm distributions, which led to the two main parameters: the equivalence diameter *d*_eq_ and the distribution width, both of which characterize the pore size distribution in the papers to a good first approach. We would like to point out that the paper structure is an open porous structure such that the definition of pore volumes and their size remains a first approach to reveal differences between the papers on the small length scale of fibers and their network.

**Figure 2:**
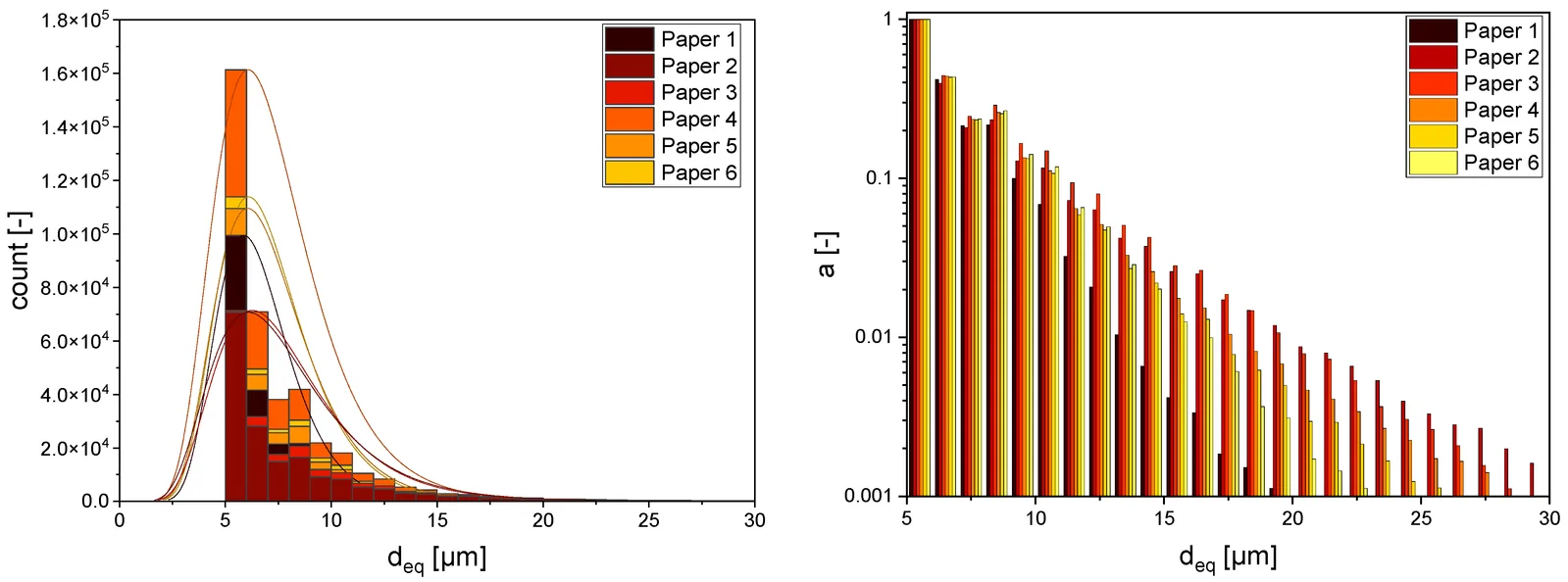
**Left**: Histograms of pore size distributions calculated from µCT images for selected substrates. Modeling of the histograms with a logarithmic normal distribution yields the solid lines. **Right**: Histograms of the portions *a*, where the counts were normalized for each paper to the fraction of smallest equivalent diameter *d*_eq_, therefore eliminating differences in the size of masks’ volumes. The differences between the substrates become evident, especially at larger pore sizes.

The histograms (Figure 2, right) show the differences in pore size distributions of the sample papers—irrespective of surface coating and paper thickness. The pore size distribution has a strong impact on the spread of the liquid ink components in the substrate after printing which will be investigated by 1D MRI and on subsequent drying. When comparing the mean equivalent diameters of the pore sizes as calculated within the model fit of the log-normal distribution to the data, the span of *d*_eq_ amounts to 5.6 µm < *d*_eq_ < 8.5 µm.

### 2.2. NMR Spectroscopy: Chemical Composition of Inks

**Figure 3:**
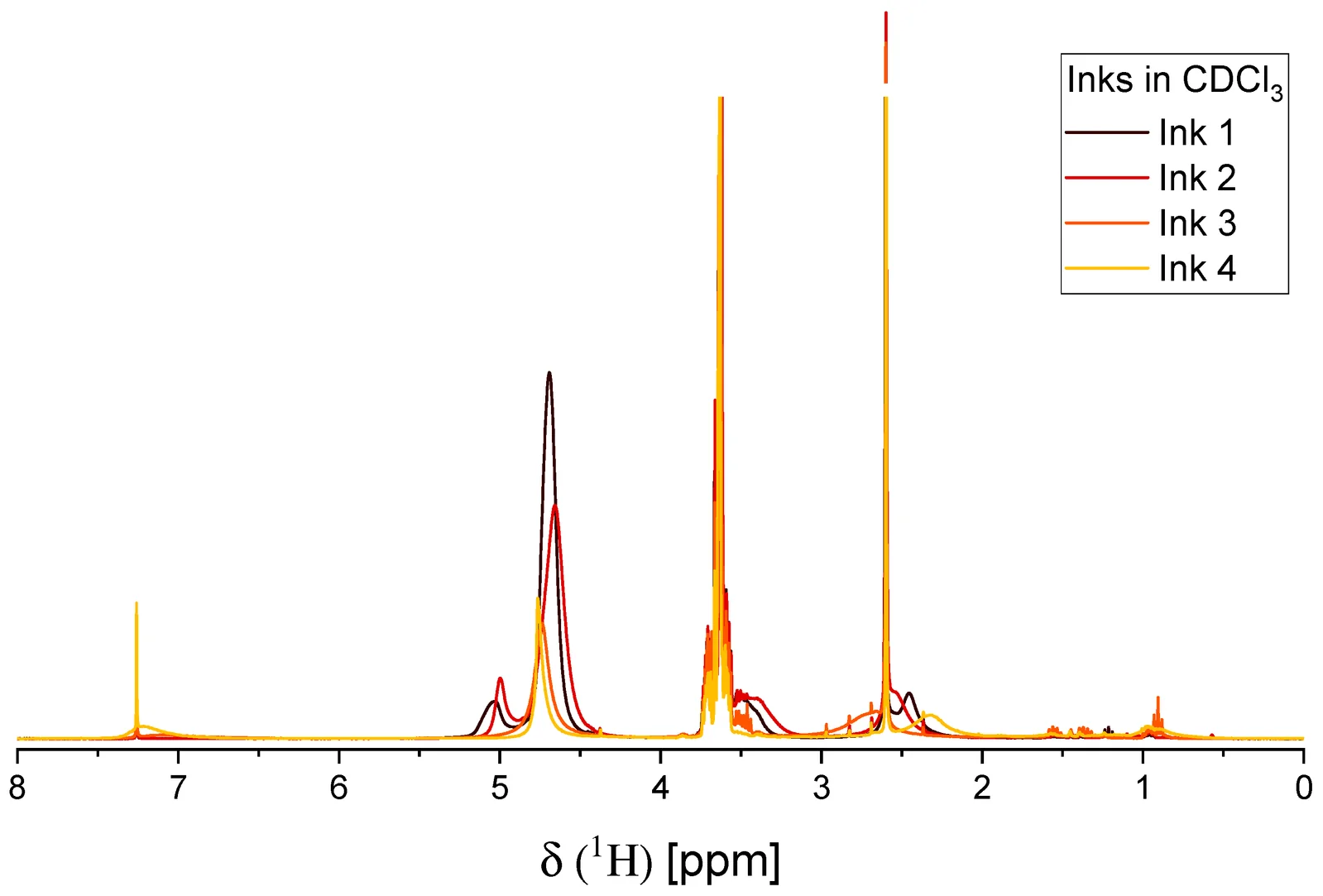
^1^H NMR spectra (300 MHz) for four inks that were diluted in CDCl_3_ (7.26 ppm) for line narrowing. In addition to the water peak around 4.6 ppm subjected to chemical exchange, the contributions from alkyl groups (0 to 2 ppm) and ^1^H nuclei near oxygen (2 to 4 ppm) appear and correspond to the functional groups of the main chemical components in water-based inks.

^1^H NMR spectroscopy was used to crudely reveal the differences in chemical composition of water-based black inks for ink-jet printing. It could be supplemented by ^13^C and 2D NMR spectroscopy, but, already, ^1^H NMR allows for a first impression on chemical composition: The inks were dissolved 1:10 in CDCl_3_ with a residual ^1^H peak at 7.26 ppm, which was chosen as chemical shift reference in all spectra. Apart from water at 4.6 ppm, several other lines show up in the spectra (Figure 3). The main components in water-based inks are—apart from water—often glycerol (4.5 ppm and lines around 3.6 ppm split up due to J-couplings), polyethylene glycol with various lines with multiplet splitting due to J-coupling between 7.2 and 1 ppm [9], and DMSO (line at 2.62 ppm). These major components can be assigned in the ^1^H spectra (Figure 3). Chemical exchange becomes obvious in the spectral region of 4.4–5 ppm, and severe line overlap needs to be taken into account also around 3.7 ppm. Additives are added to the ink’s basic recipe to improve the printing process and accuracy of the results which leads to the rather small signatures in the spectra—apart from ^13^C-^1^H splitting. Please note the different line widths, which indicates the molecular mobility of the corresponding functional groups. Broad lines are usually attributed to large molecules, especially visible in ink 1 and 4. Ink 4 shows a considerable additional contribution around 7.3 ppm, usually attributed to phenyl groups, alcohols, and other ring structures.

### 2.3. 1D MRI to Detect Penetration of Inks

**Figure 4:**
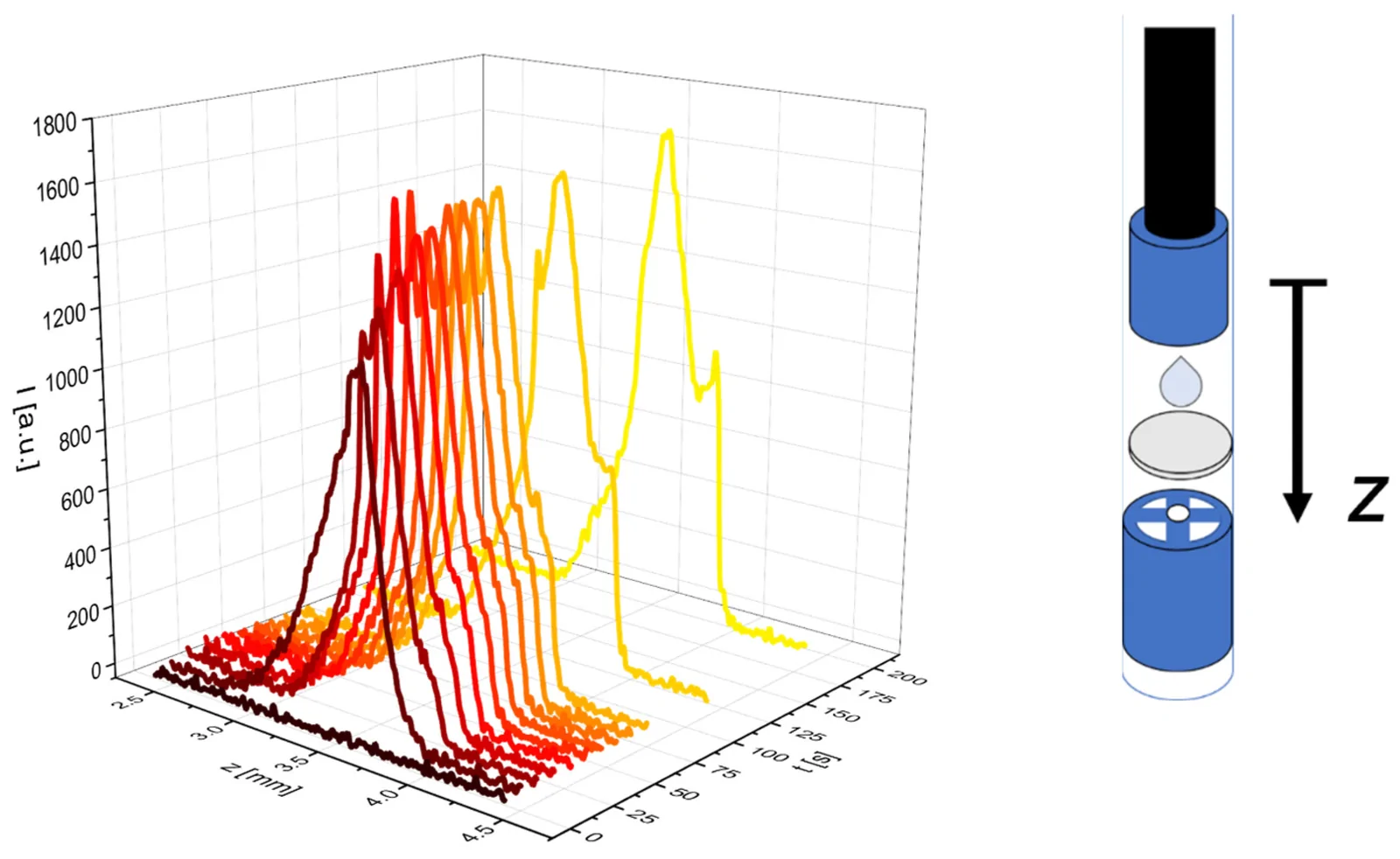
The exemplary signal intensity profiles along the *z*-axis are a function of time *t* after application of an ink droplet, so that spread and penetration of liquids can be seen immediately. The color code of the profiles reflects the increasing time (black–red–yellow). A schematic diagram of the experimental setup on the right shows the definition of the *z*-axis, the droplet of ink (light blue) and the scrap of paper substrate (grey) along with a tube for ink (black) and the supporting structure for the scrap (blue).

To investigate penetration and spread of the water-based inks in paper sheets, ^1^H MRI was applied with one-dimensional spatial resolution along the *z*-dimension (Figure 4, right). Slice selection over 4 mm was applied along one perpendicular direction, while the signal was integrally measured over the third coordinate. The time resolution amounted to 4.8 s, and the measurements of profiles started right before the application of an ink droplet to the substrate (black profile in Figure 4). The position of the paper was at *z* = 3.5–4 mm. While the time resolution of 4.8 s is not sufficiently good to reveal the droplets’ motion itself and the initial contact of ink and substrate, the penetration and distribution of the liquid in the substrate is monitored non-invasively in the porous network of fibers on longer time scales.

**Figure 5:**
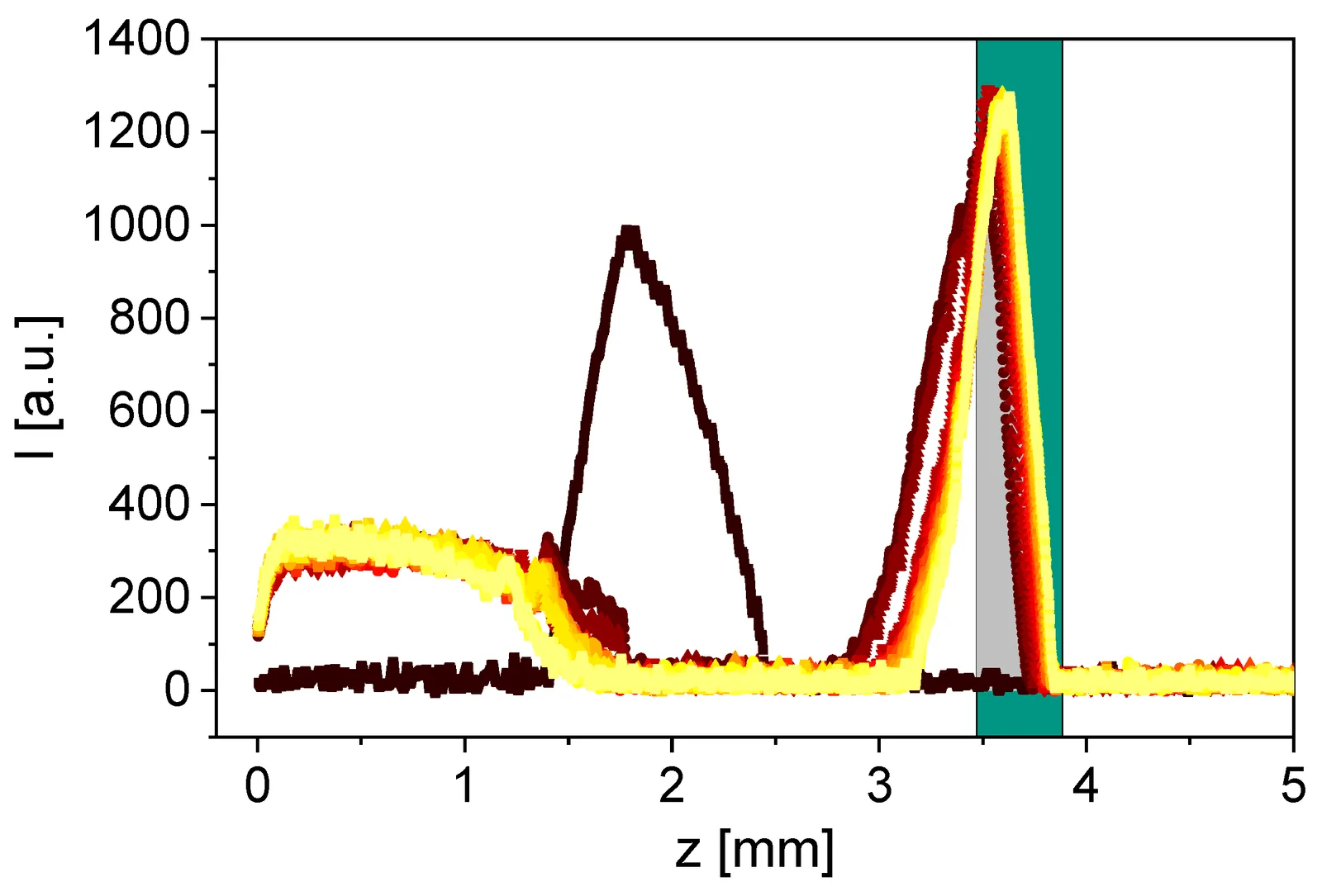
Profiles of an exemplary profiling measurement. Dark colors indicate the beginning of the measurement with the droplet at the end of the feed tube at 1.5 mm < *z* < 2.5 mm (black); light colors from red to yellow indicate progress and the end of the time resolved measurement series (see also Figure 4). The integration region, which characterizes the ink’s penetration and dispersion in the paper, is marked in green/grey.

The shape of the profiles, especially their respective maximum and width, changes with time *t* after the ink was pitching onto the paper (Figure 4 and Figure 5). The change is prominent at *z* > 3.5 mm. In Figure 5, a different combination of ink and paper inherently shows the reproducibility of the 1D profiling experiments. In this case, the droplet before “printing” is at the end of the tube (black), while paper and surrounding air show no signal.

**Figure 6:**
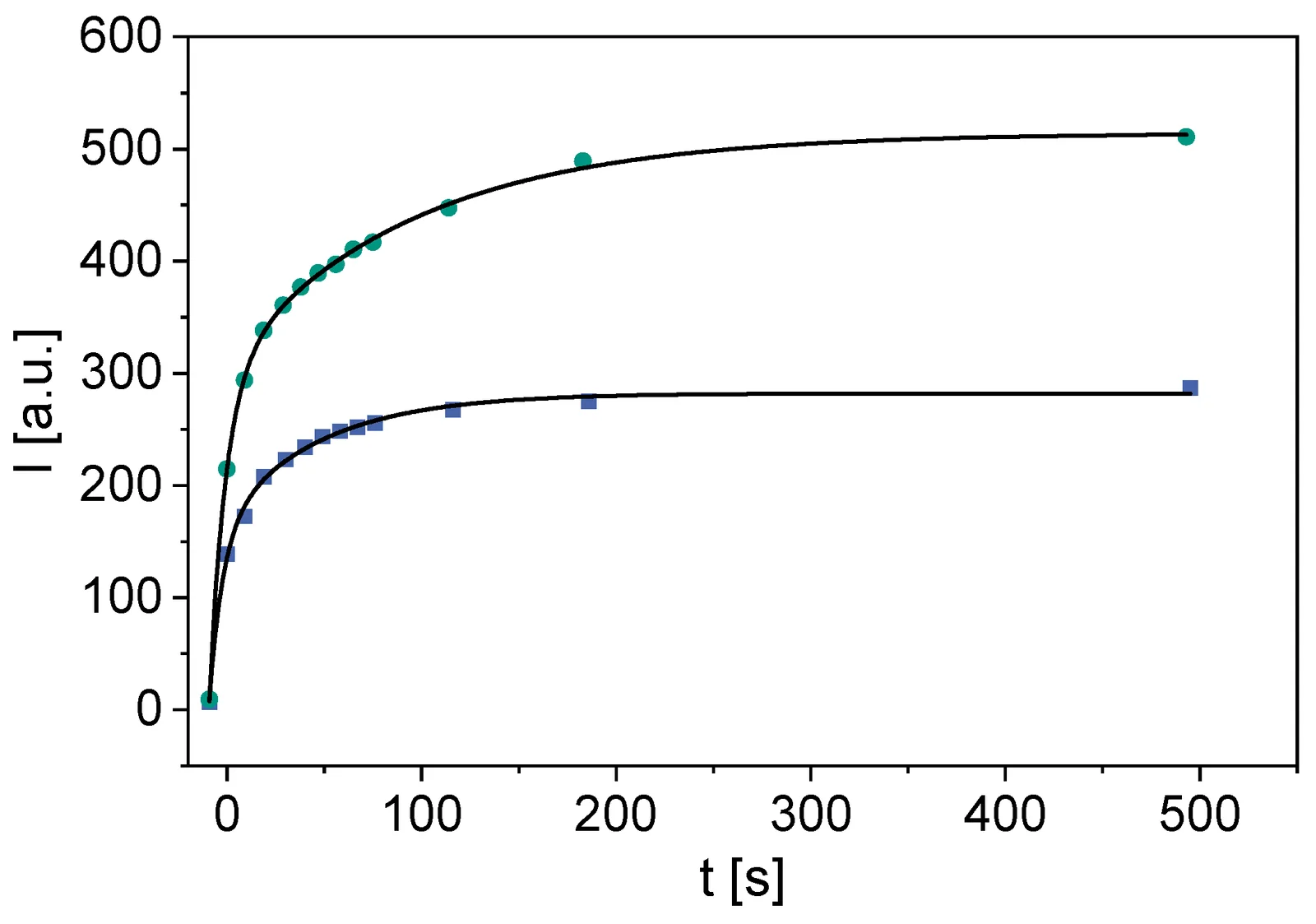
Integrals of two profiles (green and blue) as a function of the experiment time *t*, where *t* = 0 marks the moment of droplet pitching. The data are described by a double-exponential model (solid line, Equation (1)), leading to two time constants for process characterization, while the intensities at large times reflect the total amount of liquid in the integration area.

After the droplet was pitched onto the paper (colors from brown to yellow), a small amount of ink was splashed on the inner surface of the NMR tube at *z* < 1.8 mm, while a larger fraction of the liquid dropped onto the paper and penetrates with *t*. Especially at *z* > 3.4 mm, the liquid continuously spreads with increasing *t*. To reveal the kinetics of this ink’s penetration into the paper sheet, the integral from the maximum of the profile to large *z*-values around 4 mm (Figure 5, marked in green) was calculated for each time step along *t* (Figure 5), resulting in integrals as exemplarily shown in Figure 6.

The time-dependent values of the integral *I*(*t*) can be described for all measured combinations by a biexponential function:

(1) $$I\left(t\right)={I_{0}+I}_{0,1}\;\exp\left(-\frac{t}{t_{1}}\right)+I_{0,2}\;\exp\left(-\frac{t}{t_{2}}\right)$$

*I*_0_ is the long-term intensity, and the intensities *I*_0,1_ and *I*_0,2_ are the amplitudes of the two contributions. The important parameters here are the two time constants *t*_1_ and *t*_2_, which represent the time scales of the two processes revealed by fitting Equation (1) to the data of diverse combinations of ink and paper.

The time constants for the two processes are 8 s and 98 s (green symbols) and 7 s and 51 s (blue symbols), respectively, for the two exemplary combinations of ink and substrate. These two examples in Figure 6 already show significant differences in the involved time scales which characterize dispersion of the liquids in the substrates. The error when repeating the experiment 10 times amounts to 5–10%, while the measured data points can be described by the bi-exponential approach with a good numerical accuracy (in Figure 6: coefficient of determination *R*^2^ of fitting the biexponential function to the data: *R*^2^ > 0.997). The first, fast process is tentatively assigned to the pitching and initial distribution of the droplet. Due to the limited number of data points on that short time scale, the value of *t*_1_ is regarded as an order of magnitude rather than an exact, solid value. Nevertheless, the data indicate a process on that short time scale in agreement with findings on different material combinations, for example, in [3]. The second, slower process is attributed to penetrating, spreading, dispersing of ink in the substrate towards an equilibrium, followed by drying. Several data points (Figure 6) were measured on this time scale leading to a reliable value for *t*_2_.

Please note that the first contact of a droplet with the surface of the substrate is not resolved in these 1D MRI measurements, including the interaction of ink with an eventual surface coating. As the time resolution of the 1D profile measurements amounts to 4.8 s, these processes are too fast to be monitored with the presented tools.

Both processes and their time constants are specific for the combination of ink and paper under investigation. Especially *t*_2_ spans a relatively broad range (5 s < *t*_2_ < 1000 s), while 0 s < *t*_1_ < 30 s. These findings have an impact on the printing process as the time constants need to be considered in the post-printing handling of paper and during drying.

### 2.4. Correlation of Results from 1D MRI of µCT

**Figure 7:**
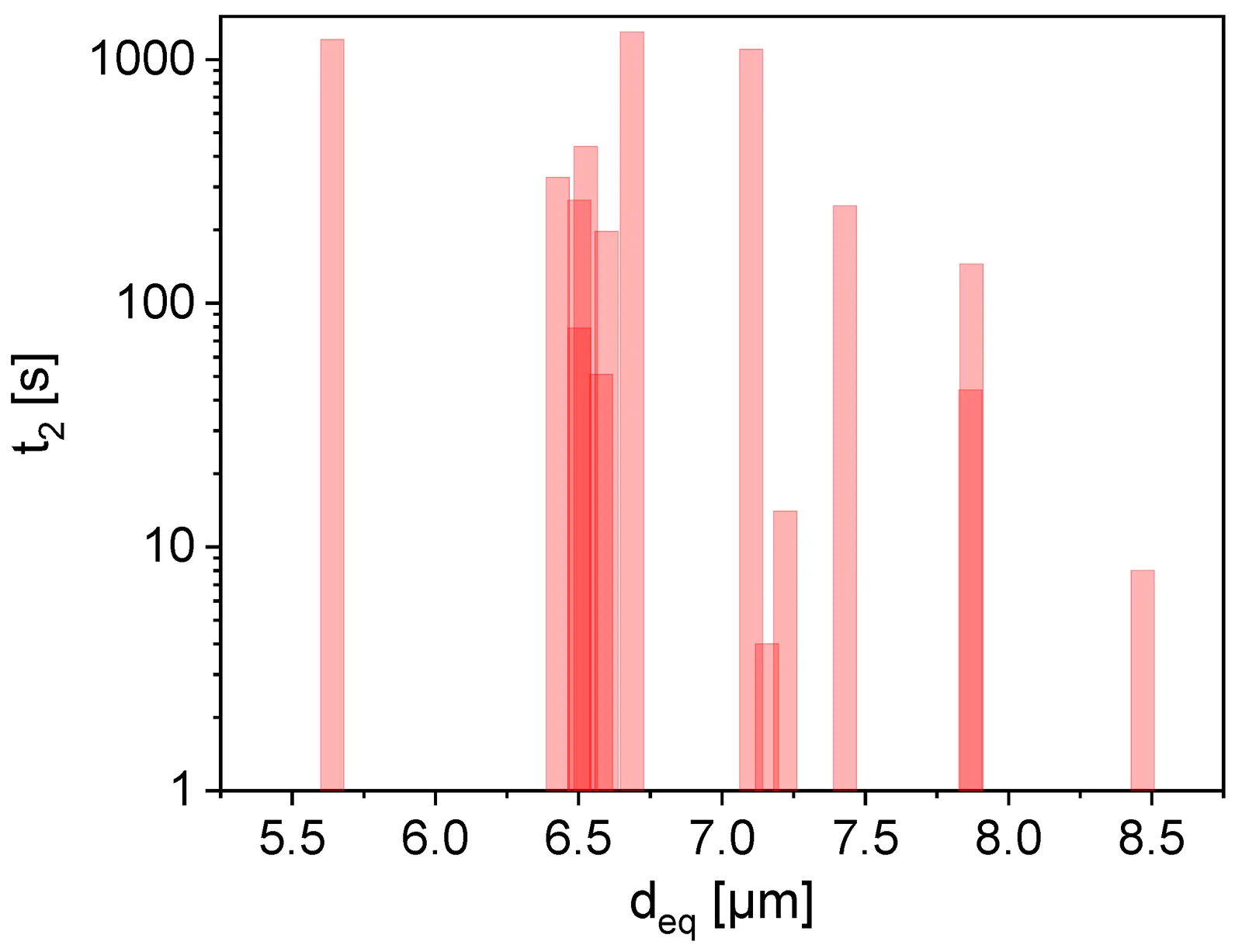
Time constant *t*_2_ deduced from the 1D MRI profiles as a function of the equivalent diameter *d*_eq_ from image analysis of 3D µCT scans on diverse substrates and one single ink. Obviously, no direct correlation of the two characterizing parameters is observed, but a large distribution width of *t*_2_.

In a first attempt, the results of 1D MRI and µCT were correlated under the assumption that the liquid dispersion in the substrates is characterized by *t*_2_ deduced from 1D MRI. Further, it is assumed that the dispersion on the longer time scale is determined by the pore size distribution in the inner of the substrates, represented by the equivalent mean diameter, which was obtained by image analysis of 3D µCT data. Please note that both data sets, the 3D scan of µCT and the 1D MRI profile, are each condensed into one single number which, as such, needs special attention and is already questionable when looking at Figure 1 and Figure 4, for example. In Figure 7, the longer time constant *t*_2_ of one specific ink applied to 15 different substrates is shown as a function of *d*_eq_. *d*_eq_ varies, as shown in Section 2.1, in the range of 5.6 µm to 8.5 µm, including commercially available papers as well as experimental papers. Thereby, larger values were measured for the commercial papers. Most interesting are the findings for *d*_eq_ around 6.5 µm: six substrates show 6.4 µm < *d*_eq_ < 6.6 µm, while *t*_2_ varies between 41 s and 440 s. This fact indicates that univariate data analysis is not an appropriate way to analyze and interpret the data. Diverse different factettes need to be considered in the characterization of the printing process. Even more, applying inks of different chemical composition on the same variety of substrates completed that picture of multifaceted influencing parameters in a printing process also on the longer time scale, which needs multivariate analysis, for example, in the form of multi-block analysis. This approach will also allow for the consideration of complete data sets, avoiding the scale reduction and simplification of data sets which was applied for Figure 7.

In addition, the new measures of characterizing the printing process, i.e., µCT, 1D MRI, and NMR-spectroscopy, will be correlated with the results of established characterization tools in printing facilities. Appropriate multivariate data processing schemes are searched for in work in progress.

## 3. Discussion

NMR in the form of spectroscopy revealed the chemical composition of the black inks for ink-jet printing. The well-established ^1^H NMR spectroscopy already allows the identification of the major chemical components and indicates the variety of additives which improve the printing results. Pigments, which are often of particulate nature, are thereby not visible in liquid-state NMR. The characterization of papers and carton boards was realized by µCT on the length scales of fibers in the range of several µm, and their network was characterized by pore size distributions. They determine—among other parameters like the interactions between fibers and ink components—the printing result, how the liquids spread in the substrate, and the drying behavior. These interactions become evident in the MRI experiments, where the interplay between ink and substrate is measured in the form of penetration and dispersion of the liquid ink in the fibrous substrates. The typical time constants of the involved processes show a large variation which reflects the microscopic properties. In summary, the single components as well as the process of printing can be characterized by the mentioned methods. The next step is to correlate the results of these analytical approaches also with the results of established characterization tools in the printing industry and research, via multivariate data analysis. Data of different numerical orders and sizes need to be considered with appropriate weighting to reveal correlations and, most interestingly, the interactions of inks and substrates to, finally, predict the printing properties and results from initial parameters like ink composition, pore size distribution, or the coating properties of paper substrates.

In addition, the application of ink droplets and the time resolution of the 1D profiling experiments are under revision to get a better comparability with the industrial process. Fast MRI pulse sequences can be optimized and designed to improve time and spatial resolution, while long living states can be investigated by multi-slicing MRI including chemical shift resolution, requiring, however, a sufficiently good spatial resolution. Severe line broadening occurred in the first experiments, which hampers attempts to get spatially resolved chemical information. Instead, NMR spectroscopy was shown to allow the differentiation of the ink’s main components, such that this approach could be widened to study diffusion properties of these components in the presence of paper fibers. Relaxation times and susceptibility differences need to be taken into account, which limit the conventional approaches.

The analytical approach sketched in this article can be used to investigate the long-term behavior of liquid distribution in substrates and, in future investigations, of drying by applying technologies like hot-air drying or drying by near-infrared radiation. Paper composition may be varied to reveal the impact of molecular interactions between substrate and ink components further and in more detail.

## 4. Materials and Methods

Different paper and carton boards for ink-jet printing were investigated, which were commercially available and hand-made by cooperation partners. They differed in fiber density as well as in surface coating. The substrate thickness was between 100 µm and 300 µm, which already is a challenge for conventional MRI—apart from the small transverse relaxation times of the fibers, which lead us to µCT to characterize the paper and carton boards.

The commercial and model inks used in this study were black and differed in chemical composition. Main components were water, polyethylene glycol, dimethyl sulfoxide (DMSO), and glycerol. In the “printing” experiments, about 2 µL of these inks were dispersed on a substrate sheet of 4 mm in diameter.

NMR spectra were measured on a 300 MHz NMR spectrometer (Nanobay, Bruker BioSpin GmbH & Co. KG, Ettlingen, Germany) equipped with a BBFO probe. The FIDs were Fourier-transformed, phased, and baseline-corrected within the software Topspin 4.0.9.

1D profiling was performed on a 200 MHz superwide-bore magnet in a MICWB40 probe with inner diameter of 10 mm connected to an HDIII console. The setup allowed us to measure 1D profiles with a spatial resolution of Δ*z* = 12 µm. The experiments were performed within the software Paravision 6, and data were subsequently processed within MATLAB^®^ R2023b and Origin^®^ 2023. Main experimental parameters are summarized in Table 1.

While NMR/MRI is most sensitive to liquids with decent transverse relaxation times, µCT is sensitive to the atomic number to the power of 3.8 and materials’ density. In the present case, the use of both analytical tools is quite comprehensive: while liquid ink and its spread in the paper substrates were investigated by methods of NMR, the paper structure was studied by µCT on the length scale of µm.

**Figure 8:**
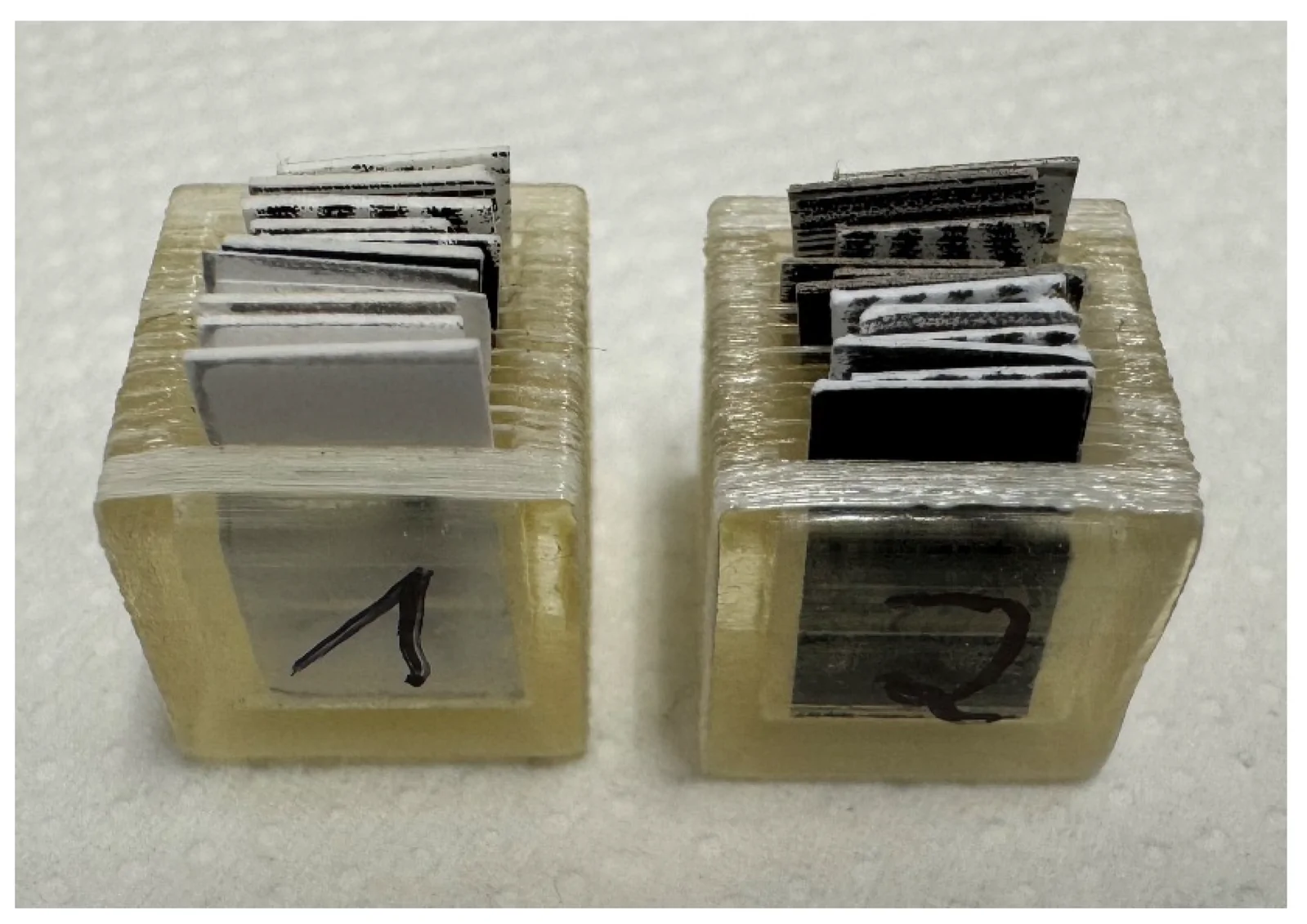
3D-printed dice were used for parallel µCT measurements of paper samples. Dimensions of the dice were 10 mm × 10 mm × 10 mm; the samples’ slots are 0.1–0.4 mm wide and show lateral dimensions of 8 mm × 7 mm.

**Figure 9:**
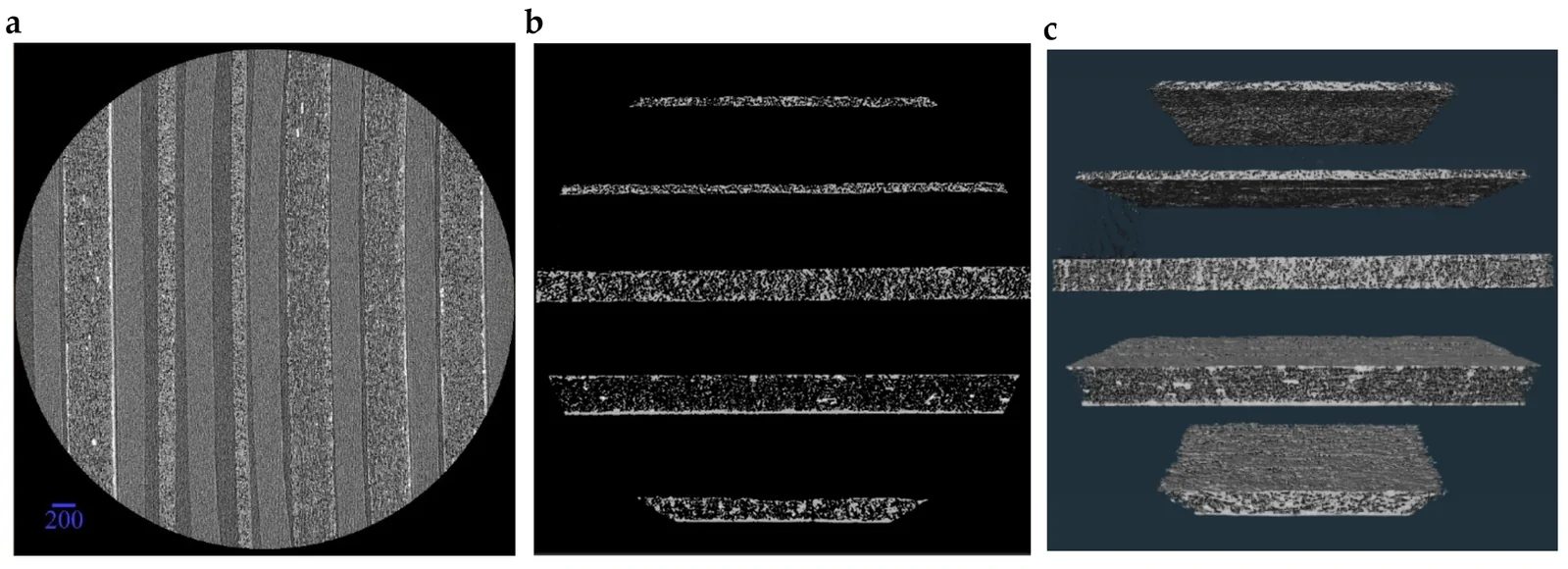
µCT images of paper stacks: (**a**) The 2D raw data show the properties of the paper samples, which differed in thickness and surface coating (length scale [µm] is indicated in blue). (**b**) Binarization makes these differences more pronounced and shows the fiber structure more obviously. Finally, volume rendering, shown in 3D in (**c**), offers the possibility of extraction of integral measures like pore size distribution. Surface coating becomes evident at reduced image noise.

µCT measurements were performed on an XRadia 520 Versa (Zeiss, Oberkochen, Germany) for resolving the structure of the paper and carton board substrates, i.e., the fibers and their network. In order to reduce the integral measurement time, paper samples were sorted into 3D-printed dice (Figure 8) to measure papers in parallel. The spatial resolution was 5 µm × 5 µm × 5 µm (Figure 9). The 2D slices of the raw 3D data sets already show pronounced differences which were worked out by dedicated image processing: The 3D data sets were processed within the software Avizo (9.1) (Thermo Fisher Scientific, Waltham, MA, USA). Data were first binarized, then subjected to volume rendering, and, finally, the already implemented pore size analysis was applied, leading to pore size distributions. These distributions were then characterized by the median of pore size given by the equivalence diameter *d*_eq_ and the distribution’s width within the model of a log-normal distribution, alternatively by the spans 25–75% and 10–90%.

## Figures and Tables

**Table 1:** Measurement and experiment parameters.

Parameters for:	Spectra	1D Profiles
Pulse sequence	Single Pulse	Spin Echo
^1^H Larmor frequency [MHz]	300	200
Echo time τ_e_ (ms)	-	6.07
Repetition time *RD* (s)	15	0.5
Number of digital points	8192	512
Number of averages (−)	32	2
Receiver gain (dB)	0.25	64
Spectral width [kHz]	4.167	150

## Data Availability

The data are available upon request from the authors.

## Abbreviations

The following abbreviations are used in this manuscript:

MRI	Magnetic Resonance Imaging
NMR	Nuclear Magnetic Resonance
µCT	Micro-computer tomography
